# A Hybrid Visual-Based SLAM Architecture: Local Filter-Based SLAM with KeyFrame-Based Global Mapping

**DOI:** 10.3390/s22010210

**Published:** 2021-12-29

**Authors:** Rodrigo Munguia, Juan-Carlos Trujillo, Edmundo Guerra, Antoni Grau

**Affiliations:** 1 Department of Computer Science (CUCEI), University of Guadalajara, Guadalajara 44430, Mexico; juancarlos_max@hotmail.com; 2 Department of Automatic Control, Technical University of Catalonia UPC, 08034 Barcelona, Spain; edmundo.guerra@upc.edu (E.G.); antoni.grau@upc.edu (A.G.)

**Keywords:** visual SLAM, filter, optimization, key-frame, hybrid, local mapping, global mapping, loop closure

## Abstract

This work presents a hybrid visual-based SLAM architecture that aims to take advantage of the strengths of each of the two main methodologies currently available for implementing visual-based SLAM systems, while at the same time minimizing some of their drawbacks. The main idea is to implement a local SLAM process using a filter-based technique, and enable the tasks of building and maintaining a consistent global map of the environment, including the loop closure problem, to use the processes implemented using optimization-based techniques. Different variants of visual-based SLAM systems can be implemented using the proposed architecture. This work also presents the implementation case of a full monocular-based SLAM system for unmanned aerial vehicles that integrates additional sensory inputs. Experiments using real data obtained from the sensors of a quadrotor are presented to validate the feasibility of the proposed approach.

## 1. Introduction

Despite the great advances reported during the last years, the Simultaneous Localization and Mapping (SLAM) problem still attracts great attention from the robotics/AI/computer vision research community since it represents a fundamental milestone in the road to developing truly autonomous mobile robots and vehicles. In this context, the visual-based SLAM systems represent an interesting sub-class of SLAM methods due to the inherent characteristics of cameras as sensors. For instance, cameras, in general, provide a lot of information about the robot environment.

Most of the approaches for implementing visual-based SLAM systems, and also visual odometry (VO) systems (e.g., [1,2]), can fall into two broad categories: (i) Filter-based methods and (ii) optimization-based methods. The first class makes use of stochastic filter-based techniques, such as the Kalman Filter [3,4] Extended Kalman Filter [5,6,7,8,9], Unscented Kalman Filter [10,11,12], Information Filter [13,14], and Particle Filter [15,16,17], for concurrently estimating the state of the robot as well as the states of the visual features composing the map of the environment. The second class of visual-based SLAM systems, which is also referred to as key-frame-based methods, decouples the robot’s localization process from the mapping process and reformulates both problems, the localization, and mapping as optimization problems. Examples of this kind of approach are [18,19,20,21,22,23,24].

In our opinion, both approaches present their own advantages and drawbacks. For instance, the filter-based SLAM methods make use of theoretically well-founded stochastic estimation tools coming from the systems and control theory. This facilitates the analysis of important system properties as the observability (e.g., [25,26,27] or the stability and convergence (e.g., [28,29], which makes it possible to design SLAM systems based on sound theoretical foundations. In this sense, optimization-based SLAM methods are in general built following a more heuristically design methodology, and therefore there is a lack of theoretical studies (math proof-type) supporting the operation of these kinds of methods. Moreover, filter-based SLAM methods are very well suited for incorporating aiding information from different sensory sources (e.g., Altimeter, IMU, range sensors, etc.) due to the data fusing nature of stochastic filters. In this sense, optimization-based SLAM methods require in general more ad-hoc solutions for incorporating data from other sensors (e.g., [30]).

On the other hand, perhaps the main drawback of filter-based SLAM methods is related to the fact that its computational requirements scale poorly as the size of the state vector increases when incorporates new map features. Although for small maps (of typically 100 features) the computational requirements are even lower for filter-based methods than those needed for optimization-based methods (see [31]), when it is necessary to build larger maps containing several hundred or even thousands of features, the filter-based methods are unable to maintain a real-time performance using consumer-degree hardware. This is when a clear advantage of the optimization-based SLAM methods becomes evident. Although optimization techniques are more computational power-consuming for small maps, their decoupled localization-mapping architecture and the use of local-optimization strategies make these kinds of methods scale computationally better when the number of map features becomes unmanageable for filter-based methods.

In this work, a new visual-based SLAM hybrid architecture is proposed which aims to take the advantages while at the same time overcoming the drawbacks of both methodologies: the filter-based SLAM methods and the optimization-based SLAM methods. The basic idea is to use each technique for what we consider is better suited according to its strengths: the filter-based technique for implementing a local SLAM process, and the optimization-based technique for building and maintaining a consistent global map of the environment.

In Section 2 the proposed architecture is presented in a detailed manner. Section 3 presents a full monocular-based SLAM system for unmanned aerial vehicles, which integrates altimeter and range measurements, as an implementation case of the proposed architecture. Section 4 presents the experimental results obtained from real data captured from the sensors of a quadrotor. Final remarks are given in Section 5.

## 2. Proposed Architecture

**Figure 1:**
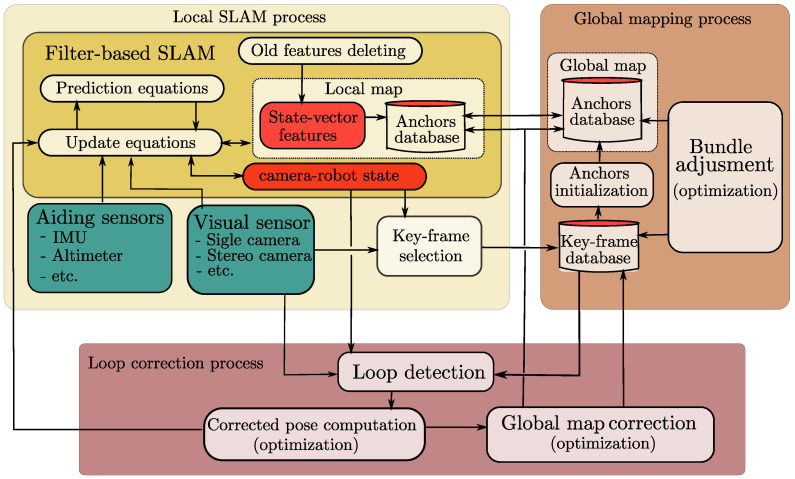
Proposed architecture.

The proposed visual-based SLAM system is composed of three processes running concurrently: (i) a local SLAM process, (ii) a global mapping process, and (iii) a loop correction process (see the Figure 1).

The local SLAM process implements a filter-based visual-based SLAM system with state vector-size bounded to maintain real-time operation. By itself, this process produces up-to metric scale (world referenced) estimates of both, the camera-robot state, and a local map of features. But in this case, since old features are removed from the vector state, to maintain real-time operation, previously visited areas of the environment can not be recognized, and thus the accumulated position drift can not be corrected by the same process alone. In this sense, the two other processes (global mapping and loop correction) will be dedicated to build and maintain a global and persistent map of the environment as well as correcting the accumulated drift when loops are detected. Some adaptations as the Key-frame selection and the use of anchors from the global map, which will be better explained later, are introduced to the filter-based SLAM method that allows interfacing with the other processes. The local SLAM process can also be conceived as a complex virtual sensor capable to provide 3D odometry information all together with visual and spatial information of the environment.

The global mapping process takes as input the Key-frames produced by the local SLAM process to create and maintain a global and persistent map of the environment. This process runs asynchronously and at a lower operation rate than the local SLAM process. This process also implements an optimization-based technique as the bundle adjustment to optimize the global map when new Key-frames are available. The map features composing the global map will be called anchors. Besides the (local) state-vector features, the local SLAM process can also make use of the (global) anchors as landmarks to correct its state. Since the global map is built upon the Key-frames produced by the local SLAM, it still will have the same drift as the position estimates of the latter.

The loop correction process is intended to minimize the drift accumulated simultaneously by the global map and the local SLAM, by detecting and closing the trajectory loops. This process runs asynchronously to the other two processes. It takes the current camera frame used by the local SLAM process and tries to associate it with previous key-frames stored by the global mapping process by means of visual descriptors. If a match is founded, indicating that this area of the environment was previously visited, then a corrected position of the camera-robot is computed. The computed camera-robot pose is used to correct the global map drift by means of a global optimization technique as the graph-SLAM, as well it is used to correct the system state estimates of the local SLAM.

In the following subsections, the proposed architecture will be described in depth: First, in Section 2.1 the local SLAM process is presented, then in Section 2.2 the global mapping process is presented, and finally in Section 2.3 the loop correction process is presented.

### 2.1. Local SLAM Process

If the following variable-size state vector x is defined:

(1) $$x={\left[\begin{array}{cccc} x_{r}^{T} & y_{1}^{T} & \cdots & y_{n}^{T} \end{array}\right]}^{T}$$

which is composed by the state of the camera-robot $x_{r}$ and the states of *n* map features $y_{i}$, i∈{1,…,n}. The *i*th feature $x_{i}$ can use any parameterization (e.g., Euclidean, Inverse-depth, etc.). And given:

(i) a continuous or discrete state-space model of the dynamics of the system:

(2) $$\dot{x}=f\left(x,u\right)\;\mathrm{or}\;x_{k+1}=f\left(x_{k},u_{k}\right)$$

where u is the input vector.

(ii) an initialization function of new map features:

(3) $$y_{new}=f\left(x_{r},z_{i}\right)$$

(iii) a series of measurements z for each sensor with measurement model $\hat{z}$:

(4) $$\hat{z}=h\left(x\right)$$

(iv) knowledge of the initial state $x_{0}$ and the initial covariance matrix $P_{0}$:

(5) $$\begin{array}{c} {\hat{x}}_{0}=E\left\{x_{0}\right\}\\ P_{0}=cov\left(x_{0},x_{0}\right)=E\left\{\left(x_{0}-{\hat{x}}_{0}\right){\left(x_{0}-{\hat{x}}_{0}\right)}^{T}\right\} \end{array}$$

where E{.} is the expected value.

Then, any general filter-based method (KF, EKF, UKF, Particle Filter, etc.) able to compute an estimate of the vector state $\hat{x}$

(6) $$\hat{x}=E\left\{x\right\}$$

can be used as a basis to implement the local SLAM process. To adapt the filter-based SLAM method to be used in the proposed architecture, the following functionalities must be implemented:

- Deleting of old features. It is well known that the computational cost of filter-based SLAM methods scales poorly as the size of the state vector increases. Therefore, “old” features that are left behind by the movement of the robot-camera must be removed from the vector state x and covariance matrix P to maintain a stable computational cost (see [32]).
- Observation of anchors. We will call *anchors* to the map features $a_{i}$ whose state and uncertainties are not stored respectively in the state vector x and the covariance matrix P. Similar to a map feature, an anchor can be any 3d static point of the environment that can be visually detected and tracked frame to frame (e.g., a corner of a desktop, or the edge of a rock). The difference to a map feature is that the anchor is considered to be a fixed reference for estimating the camera pose. That is, when an anchor $a_{i}$ is observed, the state x of the camera-robot is affected through the filter update, but the state of the anchor remains the same. Thus, the anchors act like “fixed” visual landmarks for the filter-SLAM estimation process. Anchors $a_{i}$ can use the same or different parameterization than state vector features $x_{i}$, but a new measurement model ${\hat{z}}_{a}$ (see Equation (7)) that depends on the state of the camera-robot $x_{r}$ and the constant parameter $a_{i}$ must be defined.
  (7) $${\hat{z}}_{a}=h_{a}\left(x_{r},a_{i}\right)$$
  The global map is composed only of anchors, so these will act as an interface between the local SLAM and the global mapping process. For instance, if the global mapping process modifies the position of the anchors observed by the camera, then the state x of the local SLAM will be affected accordingly.
- Key-frame selection. Key-frames $K_{j}$ are frames captured by the camera which are selected regularly among the video stream according to some specific criteria (e.g., spatial [18] or visual [19]). Each *j*th key-frame $K_{j}$ will store the camera-robot state $x_{r}$ at the moment that the frame was captured. Hereafter let define $x_{r_{j}}$ as the camera-robot state associated with the *j*th key-frame. After a Key-frame is selected it is sent to the Global mapping module to be processed.
- Position measurement update. To provide an interface with the loop correction process, a position update stage must be implemented to the local SLAM process. In this case, a position measurement model of the following type must be defined:
  (8) $${\hat{z}}_{p}={\left[x,y,z\right]}^{T}=h_{p}\left(x\right)$$
  where [x,y,z] is the position of the camera-robot expressed in the world reference frame. The corrected camera-robot positions computed by the loop correction process, are incorporated into the local SLAM by mean of this update stage.

### 2.2. Global Mapping Process

First let us define $K=\{K_{1},K_{2},...,K_{n}\}$ as the set of *n* key-frames generated by the local SLAM process and which are stored by the global mapping process. Also let us define the *global map* $A=\{a_{1},a_{2},\ldots ,a_{m}\}$ as the set of *m* anchors stored and processed by the global mapping process.

The following basic capabilities must be implemented by the global mapping process.

**Figure 2:**
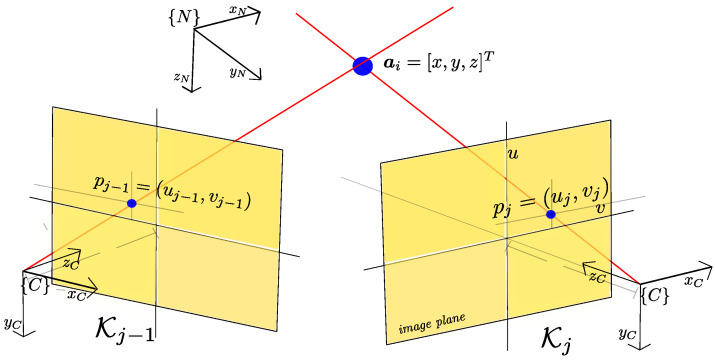
A stereo-based triangulation technique can be used for computing new anchors $a_{i}$. In this case, the camera state information associated with a pair of key-frames $\left(K_{j},K_{j-1}\right)$, and the projections $\left(p_{j},p_{j-1}\right)$ are used to compute the 3d position of anchor $a_{i}$.

- Anchors initialization. As new key-frames arrive from the local SLAM module, new anchors are computed. Each key-frame $K_{j}$ can represent a camera view since it has associate visual and spatial information. Therefore, a multiview geometry technique (e.g., [33]) can be used to compute the position of new anchors. For instance, a new subset of anchors $A_{new}$ can be computed using a stereo-based triangulation technique, where $A_{new}=f\left(K_{j},K_{j-1}\right)$ (see Figure 2).
- Bundle adjustment. In order to refine the global map, a local bundle adjustment technique is used (e.g., [34]). Assume that *n* anchors are seen (projected) in *m* key-frames, and let $v_{ij}$ be the measured projection of the *i*th anchor on the *j*th key-frame. Also let $h_{a}\left(x_{r_{j}},a_{i}\right)$ be the predicted projection of the *i*th anchor on the *j*th key-frame, where $x_{r_{j}}$ is the camera-robot state associated with the *j*th key-frame. Then, bundle adjustment minimizes the total reprojection error with respect to *n* anchors belonging to A and the *m* camera states $x_{r_{j}}$ associated with the *m* key-frames contained to K, or
  (9) $$\min_{x_{r_{j}},a_{i}}\sum_{i=1}^{n}\sum_{j=1}^{m}d{\left(h_{a}\left(x_{r_{j}},a_{i}\right),v_{ij}\right)}^{2}$$
  where d(y,x) denotes the Euclidean distance between the image points represented by vectors y and x. If the anchor *i* is not visible in the key-frame *j* then d(.,.)=0.

The operation rate of the global mapping process is not restricted by the frame-rate operation of the local SLAM process, but still, it is desirable to maintain a reasonable operation rate. When the number of key-frames and the number of anchors increases the computational cost of the bundle adjustment also increases. Therefore in practice, only a subset of the last key-frames are considered in the minimization problem, hence the name of local bundle adjustment.

### 2.3. Loop Correction Process

This process is intended to detect previously mapped areas of the environment by using visual information to minimize the position drift accumulated by both, the camera-robot state and global map estimates. In this case, the process can be implemented in fact in several ways (e.g., [35,36,37]. One of these can be for instance to apply a global bundle adjustment when the projection of old anchors, belonging to previously mapped areas, are detected and matched in recent camera frames. However in many cases, due to the great numbers of anchors and key-frames, the minimization problem involving the bundle adjustment can imply a considerable computational cost.

Based on the particularities of the proposed system architecture, the loop correction process can be implemented through the following basic functionalities:

- Loop detection. Let define F as the current frame captured by the camera, which has the current camera-state $x_{r_{F}}$ associated with it. Then, different heuristic criteria can be established, but in general, a loop is detected if enough number of visual features, belonging to F, are visually matched against some previous (old) key-frame $K_{j}$. Given the above, let defined the matched key-frame as $K_{M}$ and its associated camera-robot state as $x_{r_{M}}$.
- Corrected camera-robot pose computation. If a loop is detected, this functionality is intended to compute the corrected (relatively to the previously mapped area) camera-robot position. Let define the subset of anchors $a_{i}$ that have a projection $h_{a}\left(x_{r_{M}},a_{i}\right)$ on the key-frame $K_{M}$ as $A_{K_{M}}$, where $A_{K_{M}}\subset A$. Let define the subset of anchors $a_{i}$ belonging to $A_{K_{M}}$ that have a measured (matched) projection $h_{a}\left(x_{r_{F}},a_{i}\right)$ on the current frame F as $A_{F}$, where $A_{F}\subset A_{K_{M}}$. Moreover, let $v_{i}$ be the measured projection $h_{a}\left(x_{r_{F}},a_{i}\right)$ of the *i*th anchor on the frame F. Then, the minimization of total reprojection error with respect to *n* anchors belonging to $A_{F}$ and the camera states $x_{r_{F}}$ associated with the frame F, or
  (10) $$\min_{x_{r_{F}}}\sum_{i=1}^{n}d{\left(h_{a}\left(x_{r_{F}},a_{i}\right),v_{i}\right)}^{2}$$
  is called Perspective-n-Point (or PnP) problem (see [38]). Now let define the camera-robot state that minimizes the PnP problem as $x_{r_{c}}$. We will also refer to $x_{r_{c}}$ as the corrected camera-robot state or corrected pose. The state $x_{r_{c}}={\left[x_{cp}^{T},x_{co}^{T}\right]}^{T}$ is composed by the corrected position $x_{cp}$ and the corrected orientation $x_{co}$ of the camera-robot. When a corrected camera-robot pose is computed, a filter update is executed in the local SLAM process, to update the state of the local SLAM with $x_{cp}$ as position measurement.
- Global map correction. If a corrected camera-robot state $x_{r_{c}}$ is available, then a Graph-based SLAM technique (see [39]) can be used to accordingly correct the camera-robot states $x_{r_{i}}$ associated with the key-frames contained in K. Now, let define $x_{g}=\left[x_{r_{1}},x_{r_{2}},\ldots ,x_{r_{n}}\right]$ as the vector of parameters to be optimized by the graph-based SLAM, where $x_{r_{i}}$ describe the pose of node *i*. Note that in $x_{g}$, one parameter $x_{r_{i}}$ correspond to the state $x_{r_{M}}$ of key-frame which was matched during the loop detection. Also in $x_{g}$, the last parameter $x_{r_{n}}$ correspond to state $x_{r_{F}}$ of the camera-robot when the current frame F was captured. Let $z_{ij}$ and $\Omega_{ij}$ be respectively the mean and the information matrix of a virtual measurement between the node *i* and the node *j*. And let ${\hat{z}}_{ij}\left(x_{r_{i}},x_{r_{j}}\right)$ be the prediction of a virtual measurement given a configuration of the nodes $x_{r_{i}}$ and $x_{r_{j}}$. Finally let $e_{ij}\left(x_{r_{i}},x_{r_{j}}\right)$ be an error function that computes the difference between the expected measurement ${\hat{z}}_{ij}$ and the actual measurement $z_{ij}$:
  (11) $$e_{ij}\left(x_{r_{i}},x_{r_{j}}\right)=z_{ij}-{\hat{z}}_{ij}\left(x_{r_{i}},x_{r_{j}}\right)$$

**Figure 3:**
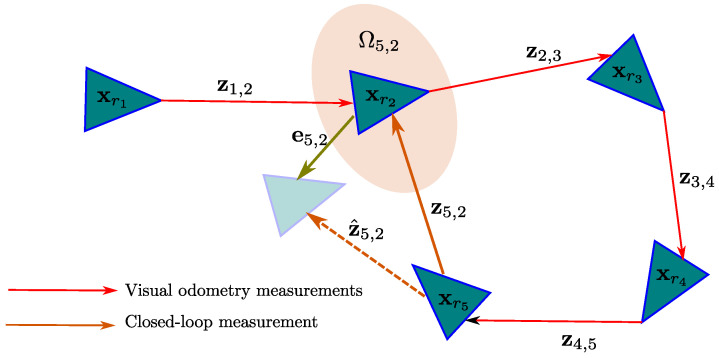
Visual representation of a Graph-based SLAM problem.

The goal of the graph-based SLAM (see Figure 3) is to found the configuration of nodes $x_{g}^{*}$ that solve the following minimization problem:

(12) $$\min_{x_{g}}\sum_{\langle i,j\rangle \in C}e_{ij}^{T}\Omega_{ij}e_{ij}$$

where C is the set of all pairs for which an observation (constraint) z exist.

For the proposed architecture, two kinds of virtual observation are defined:

- Visual odometry measurements $z_{ij}$ are defined by the relative transformation between consecutive camera-robot states, so $z_{ij}=T\left(x_{r_{i}},x_{r_{j}}\right)$, where j=i+1. In this case, there will be n−1 visual odometry measurements linking all the camera-robot states in $x_{g}$. The prediction ${\hat{z}}_{ij}\left(x_{r_{i}},x_{r_{j}}\right)$ can be set to zero ${\hat{z}}_{ij}=0$ if not visual odometry model is available (or only by simplicity).
- A single closed-loop measurement, $z_{ij}$ which is defined by the relative transformation between the corrected camera-robot pose $x_{r_{c}}$ and the state of the matched key-frame $x_{r_{j}}=x_{r_{M}}$, or $z_{ij}=T\left(x_{r_{c}},x_{r_{M}}\right)$. In this case the prediction ${\hat{z}}_{ij}$ is defined by the relative transformation between the current camera-robot pose $x_{r_{n}}=x_{r_{F}}$ and the state of the matched key-frame $x_{r_{j}}=x_{r_{M}}$, or ${\hat{z}}_{ij}=T\left(x_{r_{F}},x_{r_{M}}\right)$. In this case, for $z_{ij}$ and ${\hat{z}}_{ij}$, i=n since it corresponds to the current camera-robot state.

Each anchor $a_{i}\in A$ is linked to a specific key-frame $K_{i}\in K$ (typically the first key-frame when the anchor was initialized). If $x_{r_{i}}={\left[x_{p}^{T},x_{o}^{T}\right]}^{T}$ and $x_{r_{i}}^{*}={\left[{x_{p}^{*}}^{T},{x_{o}^{*}}^{T}\right]}^{T}$ is respectively the camera-robot state before and after of the graph-based SLAM technique is applied, then, each anchor $a_{i}$ position can be corrected with:

(13) $$a_{i}=a_{i}+\left(x_{p}^{*}-x_{p}\right)$$

Observe that when a loop is detected, the state of the local SLAM process is corrected directly by updating the state with the corrected pose $x_{cp}$, and indirectly by the observation of the corrected anchors $a_{i}$ composing the global map.

## 3. Implementation Case

In this section an implementation case of the visual-SLAM architecture proposed in Section 2 is presented. It is important to recall that the specific system described here does not represent the unique manner to implement the proposed architecture, but only represents a practical validation example among many possibilities.

The system described in this section is a monocular-based SLAM system for MAVs (Micro Aerial Vehicles). Besides the monocular camera, the system includes a barometer for integrating (absolute-referenced) altitude measurements and a range sensor for incorporating information depth. The system provides metrics estimates of the camera-robot state and the map of the environment, and it has loop-closing capabilities.

### 3.1. Local SLAM

**Figure 4:**
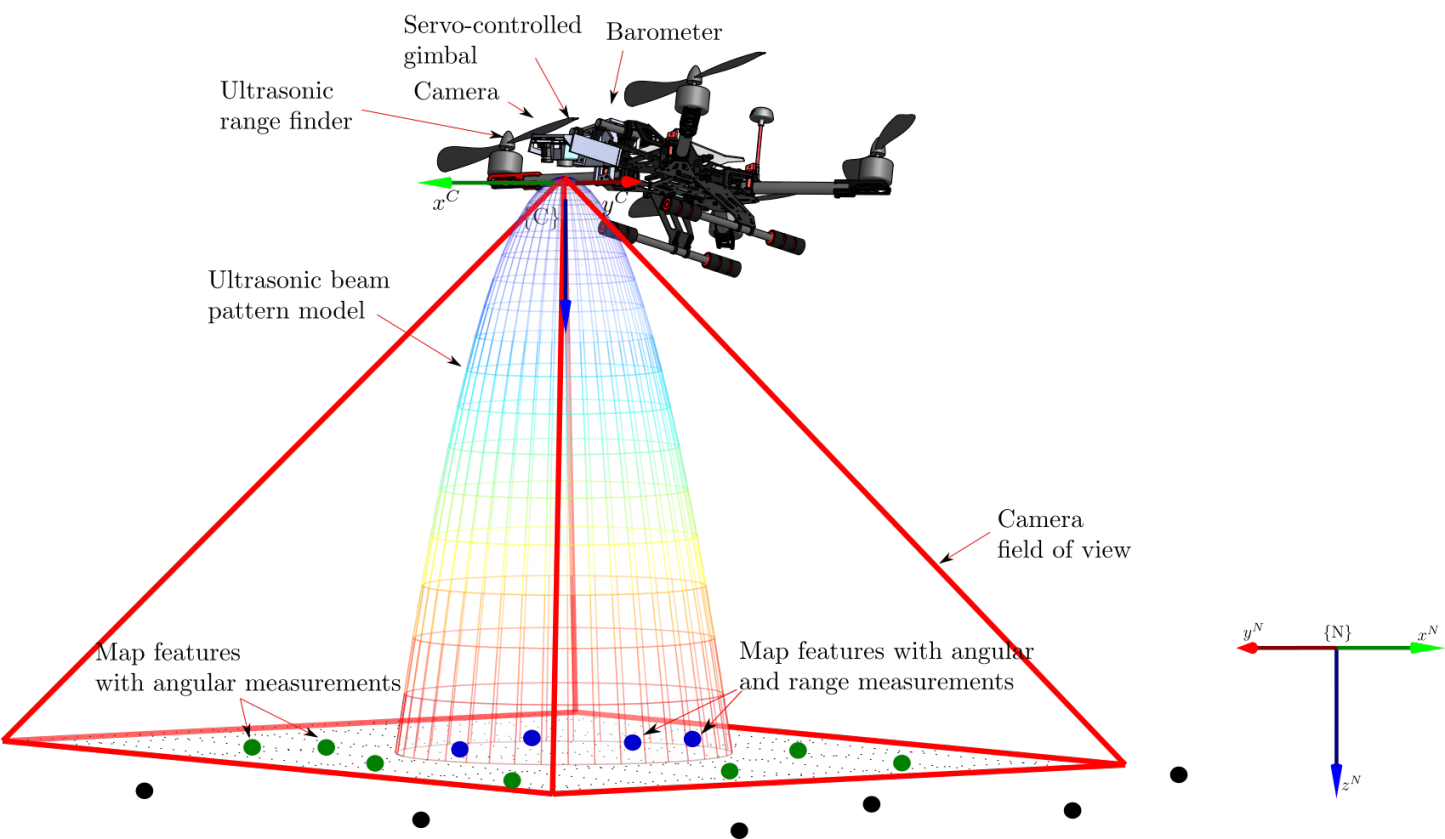
As an implementation case, a monocular-based SLAM system for a MAV equipped with a down-facing monocular camera, a barometer, and an ultrasonic range finder is presented.

The Local SLAM process is based on the authors’ previous work [40]. In this case, an Extended Kalman Filter (EKF) is used for estimating the state of a MAV equipped with a down-facing monocular camera, a barometer, and an ultrasonic range finder as well as for estimating a local map of the environment of the MAV. The camera is mounted over a servo-controlled gimbal which counteracts the changes in the attitude of the MAV. The range sensor is also mounted on the gimbal and parallel aligned with respect to the optical axis of the camera (see Figure 4).

Altitude measurements provided by the barometer are integrated for incorporating metric information into the system improving the observability of the metric scale. The range information provided by the ultrasonic sensor is integrated into the system also for improving the observability of the metric scale as well as for improving the robustness of the initialization of map features.

The elements of the state vector x defined in Equation (1) are:

(14) $$x_{r}={\left[\begin{array}{cccccc} p_{x} & p_{y} & p_{z} & v_{x} & v_{y} & v_{z} \end{array}\right]}^{T}\;y_{i}={\left[\begin{array}{ccc} p_{x_{i}} & p_{y_{i}} & p_{z_{i}} \end{array}\right]}^{T}$$

where $p^{N}=\left[p_{x},p_{y},p_{z}\right]$ represent the position of the camera-robot expressed in the navigation frame ${}^{N}$, $v^{N}=\left[v_{x},v_{y},v_{z}\right]$ represent the velocity of the camera-robot expressed in the navigation frame ${}^{N}$. And $\left[p_{x_{i}},p_{y_{i}},p_{z_{i}}\right]$ represent the position of the *i* map feature expressed in the navigation frame ${}^{N}$. Note that due to the permanent down-facing camera restriction, the problem is simplified to consider only the position estimation of the camera-robot.

The discrete state-space model (Equation (2)) is:

(15) $$\left\{\begin{array}{c} p_{k+1}^{N}=p_{k}^{N}+v_{k}^{N}\Delta t\\ v_{k+1}^{N}=v_{k}^{N}+V^{N}\\ y_{1[k+1]}=y_{1[k]}\\ :\\ y_{n[k+1]}=y_{n[k]} \end{array}\right.$$

where Δt is the time step, and $V^{N}=\sigma_{a}^{2}\Delta t$ represents an unknown linear velocity impulse with acceleration zero-mean and known-covariance Gaussian processes $\sigma_{a}$.

The model, Equation (3), for initializing new map features is:

(16) $$y_{new}=p^{N}+dR^{CN}{\left[\begin{array}{ccc} \frac{c_{x}-u^{\prime }}{f} & \frac{c_{y}-v^{\prime }}{f} & 1 \end{array}\right]}^{T}$$

(17) $$\;\mathrm{with}\;\left[\begin{array}{c} u^{\prime }\\ v^{\prime } \end{array}\right]=f_{d}^{-1}\left(u,v,k_{1},\ldots ,k_{n}\right)$$

where $R^{CN}={\left(R^{NC}\right)}^{T}$, and *d* is the approximate feature depth computed from the range sensor.

Every time that a range reading is available, new map features are initialized using the next camera frame available. First ORB keypoints [41] are detected on the frame, then a subset of strong keypoints is selected using the methodology proposed in [42]. For each strong keypoint, a new map feature is initialized in the local SLAM system state using model in Equation (16). Map features lying inside the beam pattern of the range sensor are initialized with smaller depth uncertainty than features lying outside of it. For more details about the initialization process see [40]. The corresponding ORB descriptor is stored and associated with each new map feature.

The measurement model ${\hat{z}}_{y_{i}}={\left[u,v\right]}^{T}$(Eqquation (4)), that projects a 3D map feature $y_{i}$, with position $\left[p_{x_{i}},p_{y_{i}},p_{z_{i}}\right]$, to the image coordinates [u,v] of a camera located at $p^{N}=\left[p_{x},p_{y},p_{z}\right]$ is:

(18) $$\left[\begin{array}{c} u\\ v \end{array}\right]=f_{d}\left(u^{\prime },v^{\prime },k_{1},\ldots ,k_{n}\right)\;\mathrm{with}\;s\left[\begin{array}{c} u^{\prime }\\ v^{\prime }\\ 1 \end{array}\right]=\left[\begin{array}{ccc} f & 0 & c_{x}\\ 0 & f & c_{y}\\ 0 & 0 & 1 \end{array}\right]\left[\begin{array}{cccc} r_{11} & r_{12} & r_{13} & p_{x}\\ r_{21} & r_{22} & r_{33} & p_{y}\\ r_{31} & r_{32} & r_{33} & p_{z} \end{array}\right]\left[\begin{array}{c} p_{x_{i}}\\ p_{y_{i}}\\ p_{z_{i}}\\ 1 \end{array}\right]$$

where $r_{ij}$ is the *i*-*j* element of the known (by the gimbal assumption) rotation matrix $R^{NC}$ which allows transforming from the navigation frame ${}^{N}$ to the camera frame ${}^{C}$. Let $f_{d}$ the camera distortion model depending on the distortion parameters $k_{1},\ldots ,k_{n}$, and let *f* and $\left(c_{x},c_{y}\right)$ be respectively the focal lenght and the principal point of the camera. In this work, the distortion model described in [43] is used, and the intrinsic parameters of the camera are known by calibration.

Every time a new camera frame is available ORB keypoints-descriptors are computed all over the image. Map features, that have a projection ${\hat{z}}_{y_{i}}$ over the image, are attempted to be visually matched against the ORB descriptors computed in the current frame, using a FLANN-based matcher [44]. The successful matches represent the visual measurements $z_{y_{i}}$. Moreover, a validation step (e.g., RANSAC) can be added for discarding outliers.

The measurement model for updating altitude measurements obtained from the barometer is simply ${\hat{z}}_{p_{z}}=p_{z}$. Every time a barometer measurement is available the filter is updated. For more details see [40].

The estimated state $\hat{x}$ is computed using the typical loop of filter prediction-updates steps defined by the standard EKF-based SLAM methodology (see [45,46]), along with the required adaptations described in Section 2.1:

- Old features deleting. A map feature $y_{i}$ is removed from the state vector x and the covariance matrix P, when the ratio of unmatched-matched times of a map feature is high, or number of times that it is not considered to be matched is high.
- Observation of anchors. Anchors are parameterized in the same manner as state map features:
  (19) $$a_{i}={\left[\begin{array}{ccc} p_{x_{i}} & p_{y_{i}} & p_{z_{i}} \end{array}\right]}^{T}$$
  and therefore the measurement model ${\left[u,v\right]}^{T}={\hat{z}}_{a}$ (7) is similar to (18), but in this case $\left[p_{x_{i}},p_{y_{i}},p_{z_{i}}\right]$ are fixed parameters and the jacobians does not depend on them. The local SLAM process owns a structure for storing local anchors. A local anchor is one that can be potentially projected into the current camera image-frame. Let $A_{L}$ be the set of anchors belonging to the structure owned by the local SLAM process. Anchors are added to $A_{L}$ in two ways:
  - The global mapping process copy anchors from A to $A_{L}$ that are visually linked to the current camera frame. This process will be explained in more detail later.
  - The map features contained in the state x, whose position exhibits some good degree of convergence, are removed from the EKF state and transformed into anchors contained in $A_{L}$. In this case, the following simple condition is proposed:
    (20) $$\frac{\sqrt{\sigma_{x_{i}}^{2}+\sigma_{y_{i}}^{2}+\sigma_{z_{i}}^{2}}}{\left\|p^{N}-y_{i}\right\|}<\lambda$$
    If the above criteria is met, then the transformation $y_{i}⟶a_{i}$ is carried out (simply $a_{i}=y_{i}$ ). Where $\sigma_{x_{i}}^{2},\sigma_{y_{i}}^{2},\sigma_{z_{i}}^{2}$ represent the variances of of the feature $y_{i}$ along each axis taken from the system covariance matrix, and λ is a threshold. In our implementation, a value of λ=0.1 was used.
  On the other hand, anchors are removed from $A_{L}$ in the following cases:
  - An anchor is removed from $A_{L}$ if similar conditions that those for deleting local map features are accomplished.
  - If the loop correction process has detected a loop closing condition and a corrected pose $x_{cp}$ is available, all the anchors in $A_{L}$ are deleted and replaced with visually linked anchors from the corrected global map.
  In the same manner as state features, anchors have associated an ORB descriptor. Every time a new camera frame is available the anchors $a_{i}\in A_{L}$ are projected into the image frame, and they are attempted to be matched in the same manner as the local map features to determine visual measurements of anchors $z_{a}$.
- Key-frame selection. A camera frame is selected as key-frame if two criteria are met:
  - The displacement of the camera-robot is bigger than some threshold $t_{k}$ depending on the average depth of the *n* local map features, or
    (21) $$\frac{\left\|P_{k}^{N}-P_{k-1}^{N}\right\|}{\frac{1}{n}\sum_{i=1}^{n}\left\|y_{i}-P_{k}^{N}\right\|}>t_{k}$$
    In our implementation, a value of $t_{k}=0.15$ was used.
  - A minimum number of features (or anchors) were visually matched at that frame. In our implementation, a value of 10 was used for this criteria.
- *Position measurement update.* If the loop correction process has detected a loop closing condition and therefore a corrected pose $x_{cp}$ is is available, the filter is updated in a standard manner with the measurement model (8) and the measurement $z_{p}=x_{cp}$.

### 3.2. Global Mapping

**Figure 5:**
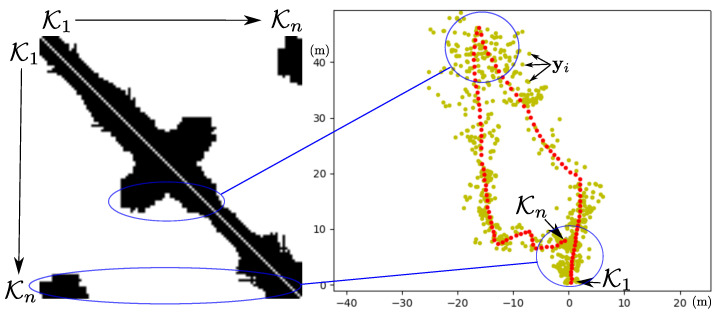
(**Left**): the plot of a matrix $V_{g}$ illustrating the visual relations between 110 key-frames taken from an actual experiment (**right plot**). White pixels indicate no visual relations. Black pixels indicate visual relation (value $w_{ij}>0$). Key-frames are indicated in red, global map anchors are indicated in yellow.

Several functionalities implemented by the global mapping process and also the loop correction process makes use of a visibility graph (see [19]), that accounts for visual relations between key-frames. Let define the visibility graph $V_{g}$ as the symmetric matrix:

(22) $$V_{g}=\left[\begin{array}{ccccc} 0 & w_{12} & w_{13} & \ldots & w_{1n}\\ w_{21} & 0 & w_{23} & \ldots & w_{2n}\\ w_{31} & w_{32} & 0 & \ldots & w_{3n}\\ : & : & : & \ddots & :\\ w_{n1} & w_{n2} & w_{n3} & \ldots & w_{nn} \end{array}\right]$$

where the component $w_{ij}=w_{ij}$ is the number of global map anchors $a_{i}\in A$, that have a projection ${\left[u,v\right]}^{T}={\hat{z}}_{a}=h_{a}\left(x_{r},a_{i}\right)$ in both key-frames, $K_{i}$ and $K_{j}$. The number of visual links of the *i*-key-frame is $w_{K_{i}}=\sum_{j=1}^{i}w_{ij}$. Figure 5 show a visual graph obtained from an actual experiment. $K_{1}$ is the first keyframe. Observe, that if the visual graph is interpreted by rows, from right to left (from recent to older key-frames), it can be inferred when the camera-robot return near to previously mapped areas.

New key-frames $K_{j}$, generated by the local SLAM process, are incorporated into the structure that stores the set of key-frames K belonging to the global map. When new key-frames are available, the following procedure is executed by the global map process:

- Computing new anchors. If a new key-frame $K_{j}$ is available and the number of visual links of the previous key-frame $w_{K_{j-1}}$ is below a threshold, then new anchors $a_{i}\in A_{new}$ are initialized by triangulation. First, visual matches are searched between the ORB descriptors of key-frames $K_{j}$ and $K_{j-1}$. Then Outliers are removed using RANSAC. If $\left[u_{j}^{\prime },v_{j}^{\prime }\right]$ and $\left[u_{j-1}^{\prime },v_{j-1}^{\prime }\right]$ are respectively the (undistorted) projection of the anchor $a_{i}$ over the key-frames $K_{j}$ and $K_{j-1}$, then the location of the anchor can be computed by triangulation as follows:
  From model (18) we have that:
  $$\left[\begin{array}{c} su^{\prime }\\ sv^{\prime }\\ s \end{array}\right]=K\left[\begin{array}{c} p_{x_{i}}\\ p_{y_{i}}\\ p_{z_{i}}\\ 1 \end{array}\right]\;\mathrm{with}\;K=\left[\begin{array}{ccc} f & 0 & c_{x}\\ 0 & f & c_{y}\\ 0 & 0 & 1 \end{array}\right]\left[\begin{array}{cccc} r_{11} & r_{12} & r_{13} & p_{x}\\ r_{21} & r_{22} & r_{33} & p_{y}\\ r_{31} & r_{32} & r_{33} & p_{z} \end{array}\right]$$
  where $a_{i}^{\prime }={\left[p_{x_{i}},p_{y_{i}},p_{z_{i}},1\right]}^{T}$, and $\left[p_{x_{i}},p_{y_{i}},p_{z_{i}}\right]$ is the position of the anchor $a_{i}$ expressed in the navigation frame ${}^{N}$. Let $k_{i}^{j}$ be the *i* row of the projection matrix K of the key-frame *j*. We have that $su^{\prime }=k_{1}^{j}a_{i}^{\prime }$, $sv^{\prime }=k_{2}^{j}a_{i}^{\prime }$, and $s=k_{3}^{j}a_{i}^{\prime }=p_{z_{i}}$. Substituting the last expression, we have that: $p_{z_{i}}u^{\prime }=k_{1}^{j}a_{i}^{\prime }$ and $p_{z_{i}}v^{\prime }=k_{2}^{j}a_{i}^{\prime }$. Considering both projections $\left[u_{j}^{\prime },v_{j}^{\prime }\right]$ and $\left[u_{j-1}^{\prime },v_{j-1}^{\prime }\right]$, of the same anchor $a_{i}$, the following linear system can be formed:
  (23) $$\left\{\begin{array}{c} p_{z_{i}}u_{j}^{\prime }=k_{1}^{j}a_{i}^{\prime }\\ p_{z_{i}}v_{j}^{\prime }=k_{2}^{j}a_{i}^{\prime }\\ p_{z_{i}}u_{j-1}^{\prime }=k_{1}^{j-1}a_{i}^{\prime }\\ p_{z_{i}}v_{j-1}^{\prime }=k_{2}^{j-1}a_{i}^{\prime } \end{array}\right.$$
  The above linear system can be solved for $a_{i}={\left[p_{x_{i}},p_{y_{i}},p_{z_{i}}\right]}^{T}$. New anchors $A_{new}$ computed by triangulation are added to the global map A
- Adding anchors computed by the local SLAM. The anchors computed by the local SLAM process $a_{i}\in A_{L}$ are added to the global map A.
- Visibility graph update. Every time a new key-frame $K_{j}$ is available, the visibility graph $V_{g}$ is updated. In this case, the visual links $w_{ij}$ are updated by taking into account the projections of the new initialized anchors over previous key-frames, and also the projections of old anchors over the new key-frame $K_{j}$.
- Bundle adjustment. First, let define the subset $K_{l}\subset K$ as the subset of *m* key-frames $\left\{K_{j},K_{j-1},\ldots ,K_{j-m-1}\right\}$ that are visually linked to the most recent key-frame $K_{j}$. A key-frame $K_{j}$ is visually linked to another key-frame $K_{j-i}$ if $w_{(j-i),j}\neq 0$. Moreover, let define the subset $A_{l}\subset A$ as the subset of *n* anchors $a_{i}\in A$ that have at least three measured projections $v_{ij}$ over three different key-frames $K_{j}\in K_{l}$. A measured projection $v_{ij}$ is determined by visually matching an anchor $a_{i}$, with predicted projection $h_{a}\left(x_{r_{j}},a_{i}\right)$, on the key-frame $K_{j}$ using ORB descriptors.
  The global map is optimized by applying local bundle adjustment (Equation (9)) to anchors $a_{i}\in A_{l}$ and setting each camera state $x_{r_{j}}$ of the key-frames $K_{j}\in K_{l}$ as fixed parameters.
- Weak anchors deleting. Anchors that can not be matched in at least three key-frames are removed from the global map to maintain only anchors with a good likelihood to be visually matched when the camera-robot revisits previously mapped areas.
- Local map anchors updating. Every time the global map is optimized by the local bundle adjustment the set of anchors $a_{i}\in A_{L}$ owned by the local SLAM process is updated. In this case, the optimized anchors $a_{i}\in A_{l}$ replaces their counterparts owned by the local SLAM $a_{i}\in A_{L}$. Moreover, the new anchors computed (and optimized) by the global SLAM process are added to the local SLAM set $A_{L}$.

### 3.3. Loop Correction

As it was described in Section 2.3, every time that it is possible, the loop correction process takes the last available frame F to detect previously mapped areas for correcting the map and camera-robot pose. In this case, the following procedure is carried out:

- Loop detection. If $K_{j}\in K$ is the most recent key-frame, and $K_{j}^{1}$ is the oldest key-frame visually linked to $K_{j}$, and $K_{j}^{2}$ is the oldest key-frame visually linked to $K_{j}^{1}$, first lets define $K_{o}\subset K$ as the subset of all key-frames not containing the key-frames visually linked to $K_{j}^{1}$ nor $K_{j}$, or: $K_{o}=\left\{K\in K:K\notin \left\{K_{j}^{2},\ldots ,K_{j}^{1},\ldots ,K_{j}\right\}\right\}$.
  The ORB descriptors, computed from the current frame F, are attempted to be matched against the ORB descriptors of key-frames $K\in K_{o}$. RANSAC is applied to remove potential match outliers. Now, let define $K_{m}\subset K_{o}$ as the subset of consecutive key-frames with at least *n* number of matches (n=15 is used by the implementation). A potential loop is detected if $K_{m}$ contain at least three key-frames. The key-frame $K_{M}\in K_{m}$ is the key-frame with the highest number of matches (see Section 2.3).
- Camera pose computation. The corrected camera pose $x_{cp}$ is computed through Equation (10), selecting the anchors $a_{i}\in K_{F}$ as it is described in Section 2.3, and with $h_{a}\left(x_{r_{F}},a_{i}\right)$ defined by the projection model (18) with $a_{i}=\left[p_{x_{i}},p_{y_{i}},p_{z_{i}}\right]$. The following considerations are taken into account for the minimization:
  - Due to the gimbal assumption, $R^{NC}$ is set as a known fixed parameter in Equation (10).
  - Due to the integration of the altimeter, $p_{z}$ (the z-axis location of the camera-robot) is set as a fixed parameter in Equation (10) equal to the current altitude camera-robot position computed by the local SLAM in $x_{r_{F}}$.
  It is important to note that the subset $K_{F}$ must contain a minimum number of four anchors to compute $x_{cp}$, but in practice, to improve robustness, a minimum number of 10 anchors is required in this implementation; otherwise, the loop closure is rejected.
  Additionally, in this implementation to improve the robustness of the solution of the corrected camera pose an additional test was considered. For this test, the anchors $a_{i}\in K_{F}$ are re-projected to the image plane through (10) using the computed $x_{cp}$. If the projection of a single anchor $a_{i}$ lies outside of the image frame then the loop closure is rejected.
- Global map correction. If a corrected camera pose $x_{cp}$ is available, then the global map (position of key-frames K∈K and anchors $a_{i}\in A$) is corrected through Equations (12) and (13) as it is described in Section 2.3. But in this case, due to that, only the position of the camera-robot must be estimated (gimbal assumption) and that the altitude computed by the local SLAM is taken to be the best estimate (altimeter assumption), the graph SLAM problem is simplified to a 2DOF (*x*-*y*) position estimation problem. Therefore, in Equation (12):
  (24) $${\hat{z}}_{ij}\left(x_{r_{i}},x_{r_{j}}\right)=\left[\begin{array}{c} p_{x}\\ p_{y} \end{array}\right]$$
  After a loop closure is carried out, the visibility graph is completely recomputed to reflect the actual visual relationships between key-frames.
- Position update triggering. When a corrected camera pose is available and the global map is rectified, a position update is triggered in the local SLAM process using $x_{cp}$ as the measurement to correct the local map accordingly to the loop close condition.

### 3.4. Implementation Notes

The implementation case of the visual-SLAM architecture proposed in Section 2 is written in c++ and makes use of the following libraries: (i) CERES [47] for solving all the minimization problems. (ii) OpenCV [48] for implementing all the image-level processing (e.g., ORB descriptors). (iii) Armadillo [49] for implementing linear algebra operations.

As it was already stated before, the proposed visual-SLAM architecture can be implemented in several manners. Meaning that also the particular implementation case, presented in this section, can be easily extended to incorporate for instance additional sensors aiding. An example o this, could be the integration of GPS measurements to the local SLAM process when they are available. Of course, the SLAM as a pure research problem aims to solve robotic navigation without depending on any external infrastructure (as the GPS). But in practical applications, the system performance will be benefited from the use of any available sensory source information.

## 4. Experimental Results

**Figure 6:**
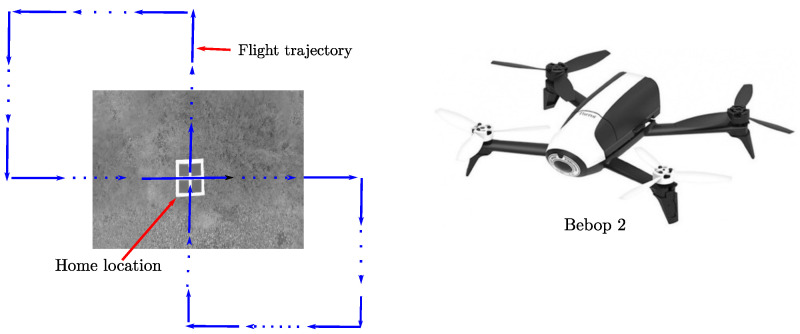
Experimental setup (**left plot**). UAV used in experiments (**right plot**).

A ground application was implemented for capturing and storing the sensors’ data obtained from a Bebop 2 quadrotor from Parrot [50] (see Figure 6, right plot). The Bebop 2 is a P7 dual-core CPU and quad-core GPU embedded system running a Linux-based OS with built in Wi-Fi, GPS and camera. It has 8GB internal flash memory, 3350 mAh battery with 25 min flying time. For our purposes, image frames from the frontal camera with a pixel resolution of 320 × 240 were captured at 30 fps. The Bebop’s camera can be set to look downwards. Moreover, altitude measurements produced by the flight controller of the Bebop, and range measurements obtained from the ultrasound sensor were captured at 5 Hz.

In experiments, the quadrotor takeoff from a specific home location, and was manually commanded to follow flight trajectories similar to the one illustrated in Figure 6, where the robot flight away from the home area and eventually returned over there. During each flight, the sensors’ data were time-stamped and stored in a data set as it was previously described. The implementation case described in Section 3 was executed in an offline manner using the captured data sets for testing the proposed visual-based SLAM approach. In this case, a major objective was to observe if the proposed SLAM system was able to close the loops and therefore to correct the error drift in both the robot position and the global map by detecting old landmarks belonging to the home area.

### 4.1. Local SLAM

**Figure 7:**
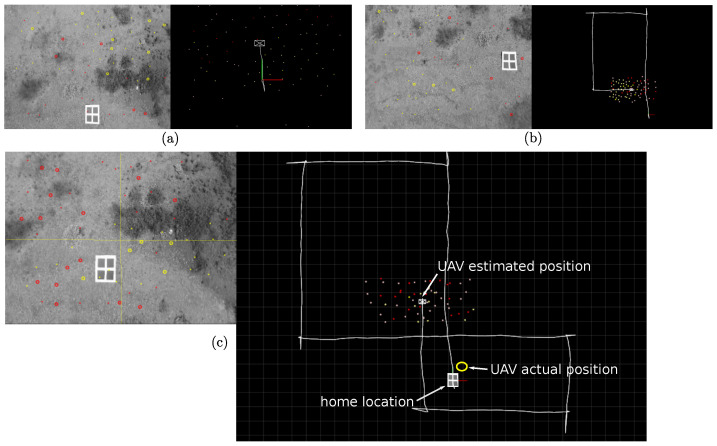
Estimates obtained from the Local SLAM process (aerial *x*-*y* view). Plot (**a**) corresponds to an initial stage of the flight trajectory, Plot (**b**) corresponds to a middle stage when the UAV is near to complete the first loop, Plot (**c**) corresponds to a final stage when the UAV has completed a second loop.

First, let analyze the estimation results obtained by the Local SLAM process. Figure 7 shows a flight trajectory computed solely by the local SLAM process. Observe that the map is always composed of visual features located near the current UAV position since they are removed from the map as the UAV moves away from them to maintain a stable computation time. In this case, it is important to note that Local SLAM can operate completely independently from the other two system process (global map and loop correction) as some kind of visual odometry and local mapping system. Of course, if previous mapped features are removed, then the Local SLAM is unable to recognize previously mapped areas (i.e., close the loop) and therefore unable to minimize the accumulated error drift in estimates. Observe in Plot (c), that by the end of the flight trajectory, the accumulated error *x*-*y* position is approximately 5 m. The above by considering that the grid in the computed scene is composed of squares of 1 × 1 m, and the home location reference measures 0.7 × 0.7 m. The UAV’s actual position at the end of the trajectory has been calculated from knowing the dimension of the home landmark (the four black-square reference), and the intrinsic camera parameters.

Table 1 shows some statistics obtained from the Local SLAM for the flight trajectory illustrated in Figure 7. Experiments were run in a laptop equipped with an Intel i7-6500U processor with 4 cores running at 2.5 GHz. The actual time duration of the flight trajectory was 86.1 s. Considering the execution times (total and per frame) is evidently that the Local SLAM process can easily meet the real-time requirements with this hardware. In this sense, it is important to note that the Local SLAM is the only process in which the execution time is constrained by the operation rate of the sensors (e.g., camera fps) to accomplish a real-time performance. Moreover, for this experiment, the use of anchors reduced the computation time by around 10 percent, without any significant differences in the estimates by using them.

### 4.2. Global Mapping

**Figure 8:**
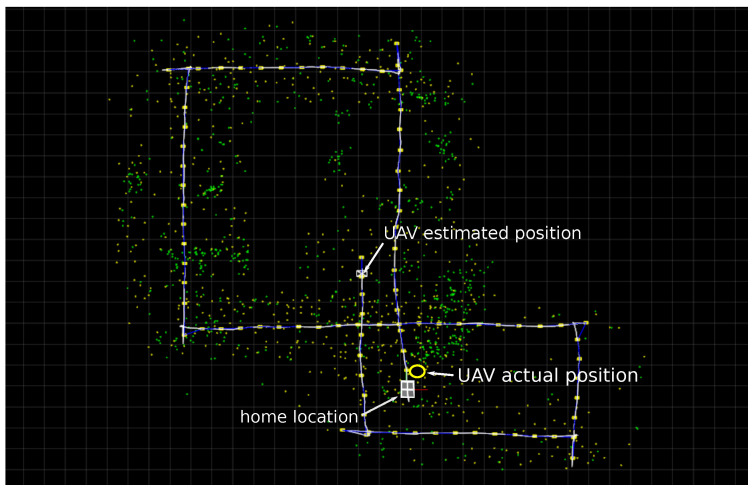
Estimates obtained from the Global Mapping process (aerial *x*-*y* view). The Global map is composed of anchors computed by the Global mapping process itself (green dots) and also by anchors computed by the local SLAM process (yellow dots).The location of the Key-frames belonging to the Global map is also highlighted along the UAV trajectory (yellow squares).

Figure 8 shows the results obtained for the same flight trajectory described in Figure 7, but in this case when the Global mapping process is taken into account. Observe that since the Global mapping process’s main task is only to construct the Global map, the same error drift exhibited by the Local SLAM remains in the estimates.

**Figure 9:**
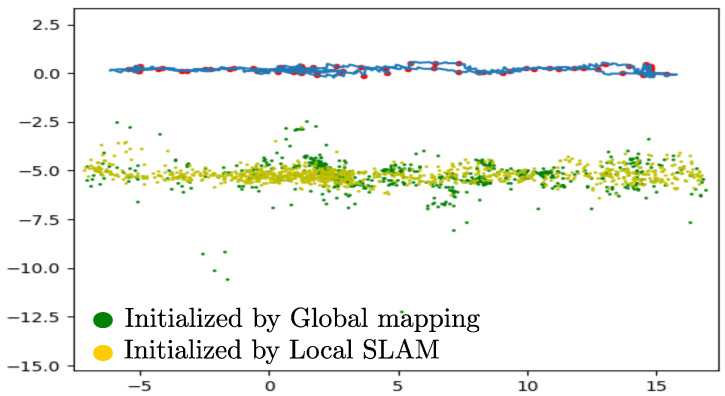
Estimates obtained from the Global Mapping process (lateral *z*-*y* view). Anchors computed by the local SLAM process are indicated in yellow. Anchors computed by the global mapping process are indicated in green.

Figure 9 shows a lateral view of the estimated global map. Since the orography of the mapped terrain is approximately flat and formed of dirt, grass, and very small bush, it gives a reference for analyzing qualitatively the depth estimates of the Local SLAM (EKF) and the Global mapping (Triangulation plus optimization). In this sense, it can be observed that the anchors computed by the Global mapping process exhibit a higher number of outliers. This result is consistent with the one presented in [31] in which filter-based SLAM methods are compared with optimization-based SLAM methods.

Table 2 and Table 3 show the statistics obtained from the global map illustrated in Figure 8. Considering this results, it is clear that the real-time performance of the global mapping process can be easily achieved by the hardware used in experiments. In fact, in future work, the unused computation time could be used for instance to perform a global bundle adjustment of the global map.

### 4.3. Loop Correction

Observing Figure 6, Figure 7 and Figure 8, it can be seen that the flight trajectory used in experiments has two potential loop detection situations, each one when the UAV passes near over the home location (at the middle and at the end of the trajectory).

**Figure 10:**
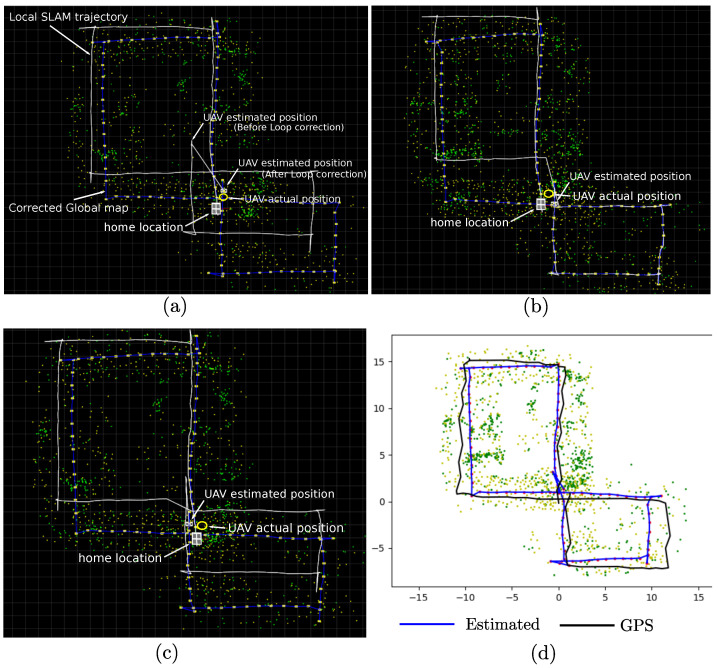
Estimates obtained from the proposed system: Local SLAM + Global mapping + Loop correction (aerial *x*-*y* view). Plot (**a**) shows a case when only the second loop was detected and corrected, Plot (**b**) shows a case when only the first loop was detected and corrected, and Plot (**c**) shows a case when both, the first and the second loop was detected and corrected. In experiments, the case (**a**) occurred more often. Plot (**d**) shows a comparison between a flight trajectory estimated from the proposed method and the one obtained from GPS.

Figure 10 shows three examples of the final estimates obtained when the Loop correction process is incorporated into the system. In this case, it is important to note that in our implementation, the detection of new visual features (ORB keypoints) over the images is carried out in a random manner. Therefore every time the algorithm is executed, it produces a slightly different estimated Local SLAM and Global map for the same flight trajectory (observe the Global maps illustrated in Figure 10). For the above reason, the chances of detecting and correcting a loop vary each time the algorithm is executed. For instance, for a sample of 100 executions of the proposed algorithm over this flight trajectory, the case (a) was obtained 55 times, the case (b) was obtained 15 times, the case (c) was obtained 18 times, and 12 times the algorithm finished without correcting any loop.

By comparing the results presented in Figure 8 and Figure 10, it can be observed that every time that the proposed system was able to detect at least one loop, the estimated Global map was considerably improved, and the final error drift in the estimated UAV position was also considerably minimized. Moreover, the correction over the Global map after loop closure can be better appreciated by comparing the Local SLAM trajectory with the final key-frames position: in Figure 8 (without loop closure) both are overlapped, in Figure 10 both differ due to the loop correction over the Global map. In this case, observe that there is a sudden “jump” in the Local SLAM trajectory every time the position of the camera-robot is corrected due to the loop closure. Figure 10 in Plot (d) also shows a comparison between a flight trajectory estimated from the proposed method (after loop-closure) and the one obtained from GPS. In this case, it is important to remark that due to its inherent sources of error, the GPS trajectory should not be taken as a perfect reference for evaluating the actual precision of the estimates. On the other hand, this kind of comparison is still relevant since it is shown that the proposed method is able to provide similar navigation capabilities to a UAV, but without the use of the GPS, which in the end is one of the major goals of the SLAM methods.

Table 4 shows the average computation time for each step of the Loop correction process. The detection of potential loops is carried out continuously at approximately at 5 Hz. The camera pose computation step is carried out only if a loop is detected and the Global map correction step is carried out only if the corrected camera pose passes the tests described in Section 3.3.

### 4.4. Comparison with an Optimization-Based Method

**Figure 11:**
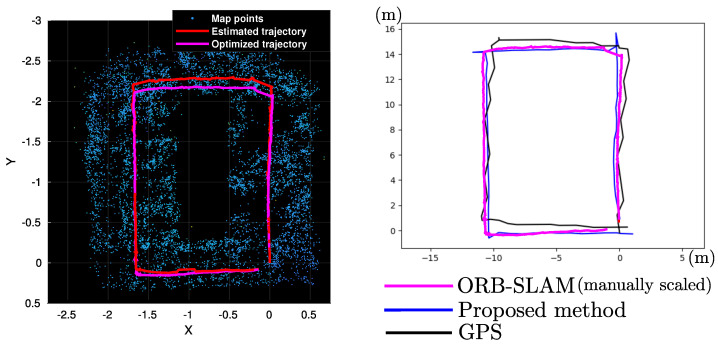
Up to scale map and trajectories computed with ORB-SLAM (**left plot**). Comparison between the trajectories obtained with (i) ORB-SLAM (manually scaled), (ii) the proposed method, and (iii) GPS (**right plot**).

Figure 11 (left plot) shows the map and trajectory computed by the well-known ORB-SLAM algorithm [19], when it is run over the first loop of the flight trajectory that was used in previous experiments. For this experiment, the official MATLAB implementation of the ORB-SLAM algorithm, provided by the Computer Vision Toolbox, was used. Since the ORB-SLAM is a purely monocular algorithm (no metric information provided by aiding sensors are considered) the map and camera trajectory is estimated only up to scale.

For this experiment, to be able to satisfactory run the ORB-SLAM algorithm over the whole trajectory, several frames had to be manually removed from the original dataset. Most of the removed frames were frames with some degree of blur and correspond to periods of fast camera movements due to sudden changes in the flight trajectory (turns in corners). Without removing those frames the algorithm always crashed due to a low number of visual features being tracked during those periods of fast movements. The above even happen when trying several parameters configurations for tracking an extremely huge number of visual features. Moreover, it is important to note that in this experiment only the first loop of the flight trajectory was used because this implementation of the ORB-SLAM algorithm only allows one close of a loop.

Figure 11 (right plot) shows a comparison between the (optimized after loop closure) trajectory computed by the ORB-SLAM algorithm, the trajectory computed by the proposed hybrid SLAM method (after loop closure), and the trajectory obtained from GPS. In this case, the ORB-SLAM trajectory was manually scaled to match the metric scale of the other two trajectories.

Table 5 shows some statistics obtained from both methods: the ORB-SLAM and the proposed method. It is very important to consider that the numbers expressed in this table are included only for reference and should be not taken as a direct measurement of the performance of both methods. For instance, MATLAB implementations usually run much slower than C++ implementations. Moreover, there are so many structural differences between methods, that make it difficult to carry out an in-depth comparative study. On the other hand, what this simple experiment suggests is that a visual SLAM system implemented with the hybrid architecture proposed in this work is able to provide similar (but metric-scaled) estimates that those obtained by a state-of-art purely visual-based SLAM method.

### 4.5. Other Flight Trajectories

**Figure 12:**
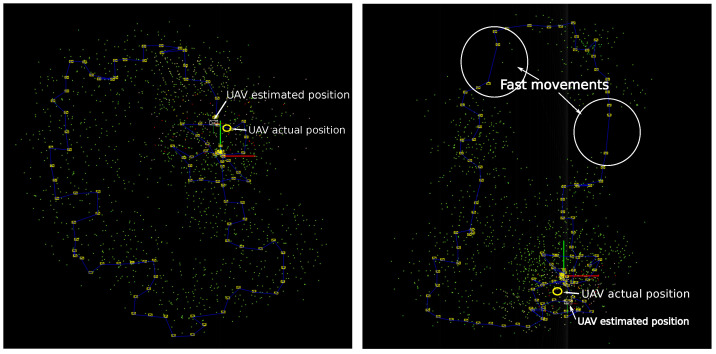
Results obtained from two different flight trajectories with a closed-loop.

The SLAM system described in Section 3 can not only be applied to simple flight trajectories as the one used in previous experiments. Figure 12 shows the results obtained from two different flight trajectories with a closed-loop. Observe that both trajectories present several changes in direction which in turn corresponds to attitude changes of the drone. Moreover, observe in the right plot that the flight trajectory includes a couple of periods where the drone moves too fast. During those periods, the images captured from the camera are very blurred, and therefore, the tracking of image features becomes unreliable (observe the absence of map features and key-frames in those areas). The proposed method is able to cope with this situation because during short periods where visual information is unavailable, the estimates are computed from the prediction stage of the filter, and the remaining available sensors updates (e.g., altimeter). In both cases, after the loop closure correction, the error in the estimated position is less than 0.5 m. To have a reference for the metric scale of the estimates consider that the (green-red) home axis has a length of one meter.

## 5. Conclusions

The experimental results previously presented show that the monocular-based SLAM system for UAVs described in Section 3, which was implemented following the proposed architecture described in Section 2, is able to estimate the state of the robot by using only onboard sensors while at the same time building and maintaining a global map of its environment. The system was able to considerably minimize the error drift in position by detecting closing the trajectory loops. It also was shown that the SLAM system can easily achieve real-time performance using consumer-degree hardware.

It is important to remark again that this monocular-based SLAM system for UAVs represents only a case of many possible ones of implementations of the visual-based hybrid architecture proposed in this work. For instance, a SLAM system for ground vehicles that makes use of an omnidirectional camera or stereo camera as a principal sensor and which uses a Unscented Kalman Filter for implementing the local SLAM process can be also implemented based on the proposed hybrid architecture. In this sense, future work could include development and experimentation with different visual-SLAM applications using the proposed architecture. It could also include testing the performance of the monocular-based SLAM system for UAVs presented in this work in an online context (not running in a data set), and perhaps using the estimated state as the feedback signal of an autonomous flight control system. Moreover, in future work, the path-tracking accuracy of the mobile robot could be evaluated based on the information proposed by the onboard sensors as in [51].

## Figures and Tables

**Table 1:** Statistics of the Local SLAM process. In this table, feats:I/D is the relation between the total number of initialized and the deleted EKF features, feat/frame is the average number of features per frame, anchors:I/D is the relation between the total number of initialized and the deleted anchors, time(s)/frame is the average computation time per frame, and total time(s) is the total computation time.

	Feats:I/D	Feat/Frame	Anchors:I/D	Anchors/Frame	Time(s)/Frame	Total Time(s)
No anchors	17,226/17,146	83.2 ± 7.03σ	0	0	0.0118 ± 0.0025σ	30.77
With anchors	11,163/11,134	30.49 ± 9.42σ	1496/1436	60.4 ± 11.82σ	0.0106 ± 0.0023σ	27.54

**Table 2:** Statistics of the Global mapping process. In this table, number KF is the total number of Key-frames contained by the global map, anchors:I/D is the relation between the number of initialized and deleted anchors carried out by the global mapping process, anchors:GM is the number of anchors composing the global map that was initialized by the global mapping process, anchors:LS is the number of anchors composing the global map that was initialized by the local SLAM process, anchors:total is the total number of anchors composing the global map.

	Number KF	Anchors:I/D	Anchors:GM	Anchors:LS	Anchors:Total
Global Map	99	2299/1624	675	1008	1683

**Table 3:** Computation times of the Global mapping process. In this table, n updates is the number of updates carried out by the global mapping process (an update includes the execution of all the steps described in Section 3.2), KF optimized per update is the average number of key-frames optimized by the local bundle adjustment step, Time per update (s) is the execution time per global mapping update, Total time (s) is the total execution time of the global mapping process during the flight trajectory.

	n Updates	KF Optimized per Update	Time per Update (s)	Total Time (s)
Global Map	97	17.4 ± 8.1σ	0.082 ± 0.032σ	8.001

**Table 4:** Computation times of the Loop correction process. In this table, loop detection is the average execution time needed for detecting potential loops, Camera pose comp is the average execution time needed for computing the corrected camera pose, and Global map correction is the average execution time needed for correcting the global map.

	Loop Detection (s)	Camera Pose Comp. (s)	Global Map Correction (s)
Loop correction	0.183 ± 0.161σ	0.382 ± 0.021σ	0.379 ± 0.010σ

**Table 5:** Statistics of the comparison between the ORB-SLAM and the proposed Hybrid method. In this table, n map feats is the number features/anchors contained by the map, n key-frames is the number of key-frames, and Execution time is overall execution time.

	n Map Feats	n Key-Frames	Execution Time (s)
ORB-SLAM (MATLAB)	8704	274	331.72
Hybrid SLAM (C++)	846	58	20.01
